# The purity of sacred lotus: superhydrophobic self-cleaning plant surfaces and the consequences revisited

**DOI:** 10.1007/s00425-026-04937-9

**Published:** 2026-02-22

**Authors:** Wilhelm Barthlott

**Affiliations:** Bonn Institute for Organismic Biology BIOB, University of Bonn, Meckenheimer Allee 170, 53115 Bonn, Germany

**Keywords:** Bionics, Cuticle, Material science-*Nelumbo*, Water repellency

## Abstract

**Main conclusion:**

Superhydrophobicity and self-cleaning (Lotus Effect) came only in focus of research after 1997. Botanic systematic studies led to a paradigm shift in materials science and numerous technical applications. However, physics behind it is still not fully understood. Details on the discovery, consequences, and open questions are presented.

**Abstract:**

Extreme water repellency (superhydrophobicity) is a feature of many biological surfaces from terrestrial cyanobacteria to green plants and animals. The initially controversially discussed publication “*Purity of sacred Lotus or escape from contamination on biological surfaces*” (Planta 1997) showed that defined hierarchically structured superhydrophobic surfaces reduce the adhesion of pathogens and particles as defense mechanism. The technical applicability was indicated, and the publication initiated about 2000 publications annually and numerous applications in our daily life. Although cuticular plant surfaces are probably the largest homogenous interfaces on our planet, they came very late in the focus of research. Functional principles, occurrence of self-cleaning biological surfaces, the physical background, patenting consequences, and open questions are discussed.

## Introduction and research background

Despite a long history of botanical research, surfaces and wax structures came late into focus. Data in textbooks were limited and waxes were regarded as protection against water loss through the cuticle. In physics, the term “superhydrophobicity” is used for extreme water repellency since the 1990s. Surfaces as boundary layers are the crucial interface for the interactions between solids and their liquid or gaseous environments. They are easily accessible and their function seems to be obvious, but the physics of their interactions is complex—a proverbial sentence is attributed to Wolfgang Pauli *“God created the solids, but the devil made their surfaces”*.

**Fig. 1:**
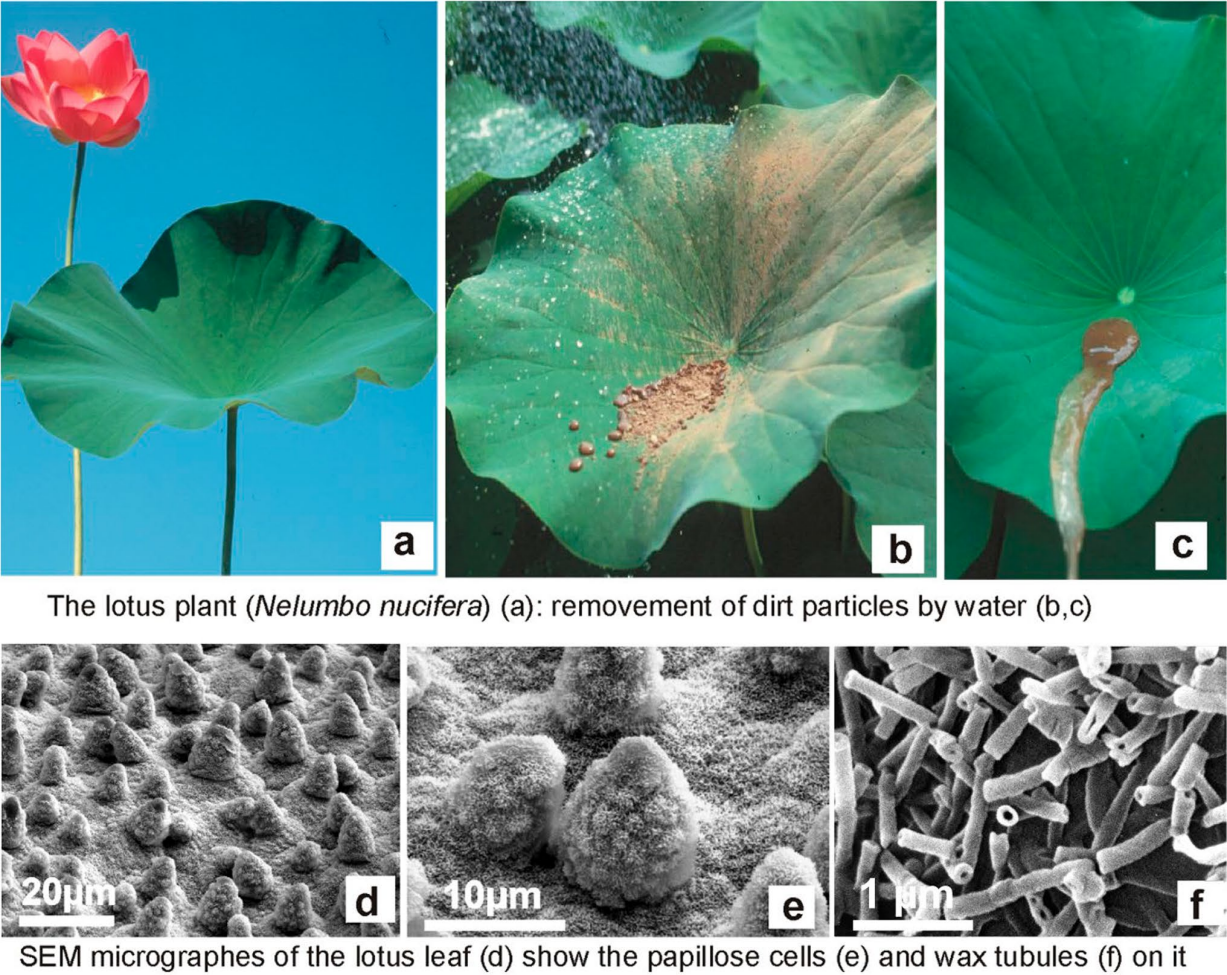
**a**–**c** Superhydrophobic and self-cleaning surface of Lotus (*Nelumbo nucifera*). Flowering Lotus plant (**a**), Lotus leaf contaminated with clay (**b**), and its removal by water (**c**). **d**–**f** SEM micrographs showing the Lotus leaf hierarchical surface in different magnifications: the papillate epidermis (**d**), single cell papillae covered by epicuticular nonacosanol tubular crystals (**e**), and high resolution of the nonacosan-10-ol tubular crystals (diameter of a single crystal 110 nm, **f**). Figure 21 from Barthlott et al. (2017), https://link.springer.com/article/10.1007/s40820-016-0125-1

**Fig. 2:**
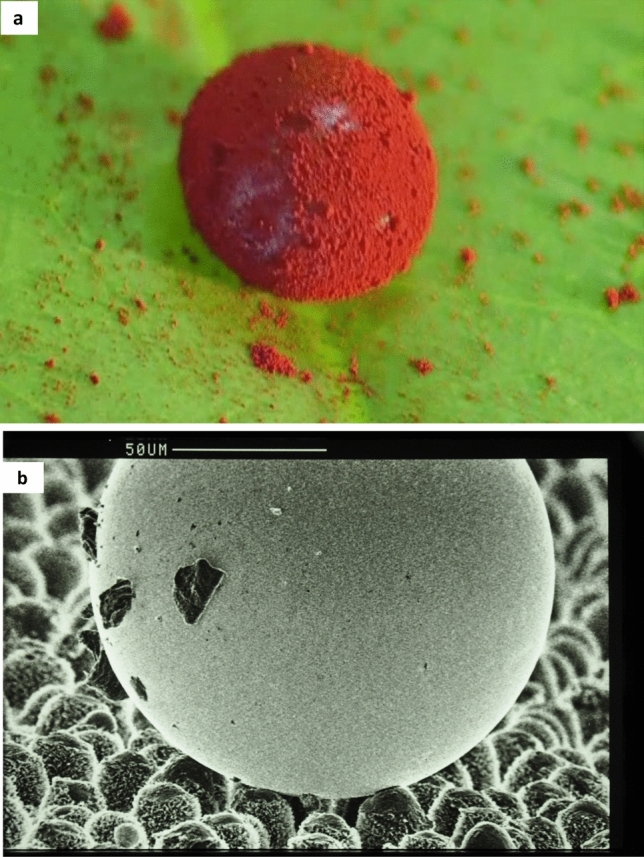
Lotus Effect: self-cleaning through droplets. **a** A waxy hydrophobic Lotus (*Nelumbo*) leaf contaminated with red Sudan IV-powder. The hydrophobic powder is removed by a water droplet forming a liquid marble. Picture from Barthlott (1992). **b** Small mercury droplet on the leaf surface of taro (*Colocasia esculenta)*: Contaminating particles adhere to the surface of the droplet and are removed from the leaf when the droplet rolls off. Bar = 50 µm. Figure 7 from Barthlott and Neinhuis (1997)

Almost 400,000 different species of land-living plants exist with an overwhelming diversity of surface structures evolved over half a billion years via mutations (trial) and selection (error) as defense mechanisms against contamination with solids (Figs. 1, 2) or pathogens (survey in Barthlott et al. (2017, 2022)). Evolution sometimes results in unexpected optimized solutions which inspire biomimetic applications by engineers and materials scientists.

The earlier lack of information on the fine structure of plant surfaces covered by self-assembling wax crystals can be explained by the microscopic approaches used. It is difficult to analyze these surfaces by light microscopy (LM) and transmission electron microscopy (TEM), because of the limited resolution of LM and because the wax structures are removed during the preparation with organic solvents. Further research was connected to the availability of scanning electron microscopes (SEM) since the late 1960s. Earliest publications on the SEM of epicuticular wax covers were published in Planta (Rentschler 1971), and Baker and Holloway (1971) provided the first data on the ultrastructure.

My research began in 1971 as a doctoral student in Heidelberg at the Institute for Systematic Botany and Plant Geography, working on a taxonomic thesis supervised by Werner Rauh. Together with Rauh and the postdocs Nesta Ehler and Rainer Schill, we applied to the DFG for a Cambridge Stereoscan SEM for taxonomic pollen studies, which was established in 1972. However, I was more intrigued by the unexpected cuticular surface structures and their possible functions, resulting in many publications between 1973 and 1977 (survey in Barthlott 1981).

**Fig. 3:**
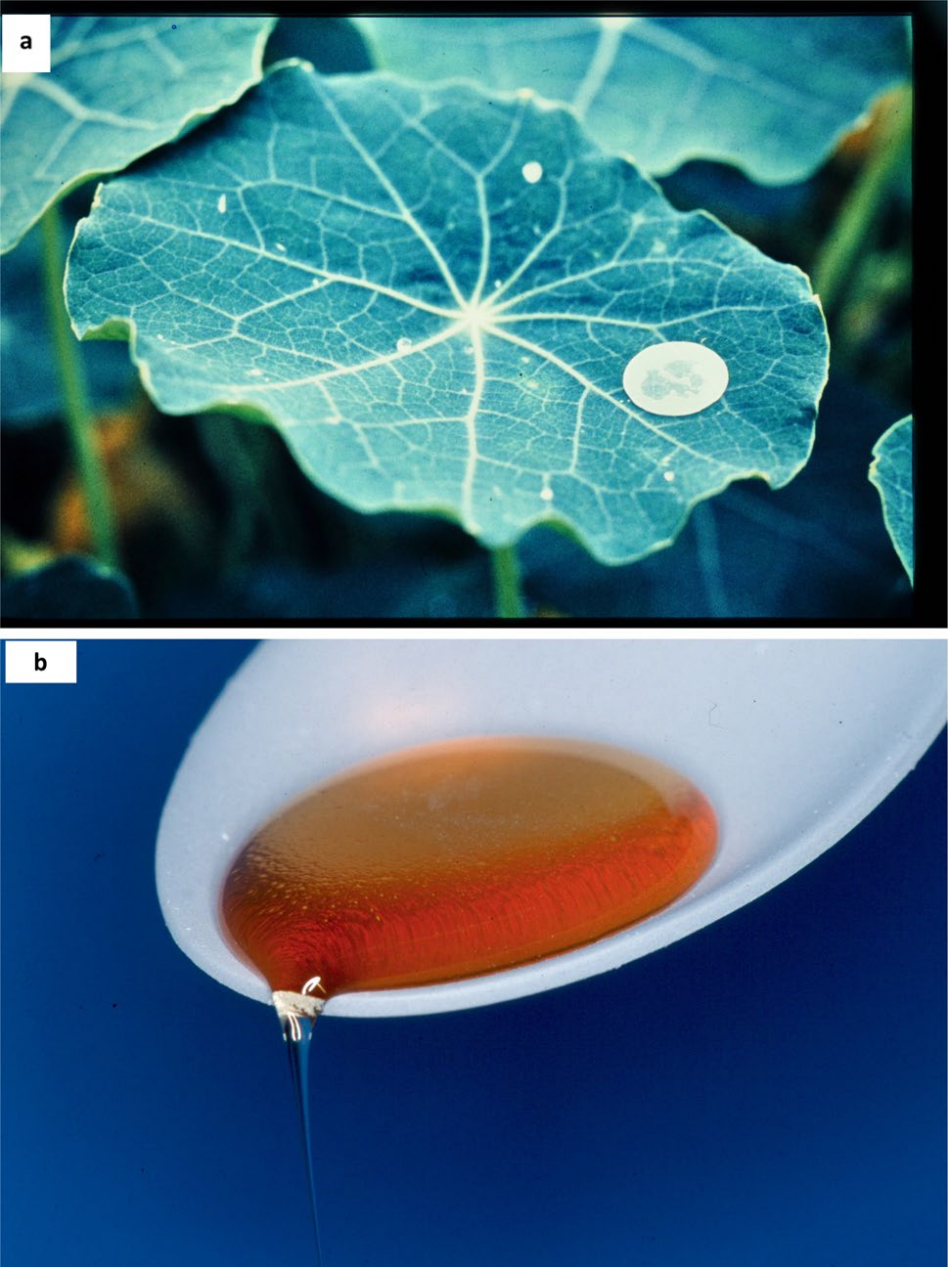
History of self-cleaning surfaces. **a** Superhydrophobic structured leaf of Indian cress (*Tropaeolum majus*) with an attached flat circular microscopic glass cover slide exposed to outdoor contaminations in the Botanical Garden Heidelberg in 1974. Leaf surface and glass cover were differently contaminated after 1 month of exposure. Photograph from Barthlott (1974). **b** A biomimetic hierarchical-structured superhydrophobic spoon with sticky honey running off without leaving any residue. That first technical Lotus prototype, produced two decades later 1994 in Bonn, finally led to industrial co-operations. Picture available at https://www.flickr.com/photos/lotus-salvinia/12475878995/in/album-72157640870446734

At that time, plant surface waxes were seen exclusively as a barrier against water loss—which was well known for the intra-cuticular waxes (Riederer and Schreiber 2001). However, this did not explain the diversity and complexity of superimposed epicuticular crystalline wax structures as the outermost interface. I observed in 1974 in the Botanical Garden that water-repellent leaves were usually less contaminated in contrast to wettable surfaces. First systematic contamination experiments were carried out using the superhydrophobic leaves of *Tropaeolum* (Fig. 3a), and we identified a self-cleaning mechanism to avoid contamination, e.g., by dust and fungal spores, as an important function of these surfaces (Barthlott and Ehler 1977). This comprehensive monograph described for the first time the principle of superimposed hierarchical structures, their diversity, and their extreme water repellency connected to self-cleaning. The publication and a follow-up survey (Barthlott 1981) remained almost unnoticed by the scientific community. At that time, we were convinced that the obvious self-cleaning of these surfaces was a phenomenon already known in physics and materials science and only new to botanists. Only later I became aware that it was overlooked, and introduced the short term “Lotus Effect” (Barthlott 1992).

The name Lotus can cause confusion. The non-wettable Sacred Lotus [*Nelumbo nucifera,* Nelumbonaceae (Fig. 1a)], is native to subtropical Eurasia, the ssp. *aurea* to E North to Central America (Borsch and Barthlott 1994). Problematic in publications, is the confusion of *Nelumbo* with the wettable water lily *Nymphaea* (Nymphaeaceae), which is also often called colloquially Lotus: the “White Lotus” (*Nymphaea lotus*) and the “Blue Lotus” (*Nymphaea nouchali* var. *caerulea*).

The Heidelberg research was continued in Bonn from 1985 on with Martin Wolter, Christoph Neinhuis, Kerstin Koch, and Matthias Mail and others (see references). Our applications for funding were rejected, and we relied on grants from the Academy of Science in Mainz. Finally, the German Federal Research Ministry BMFT provided support in 1989, but very restricted within a ‘Forest Decline’ program, a topic fashionable at that time. After we produced the first biomimetic technical prototype in 1994 (Fig. 3b), decisive funding came from the German Federal Environmental Foundation DBU.

Like obtaining funding, it was initially difficult to publish the work. Reasons for four rejections by peer-reviewed journals 1994–1995 were statements like “*poor and only descriptive, without physical modelling*” or *“no new finding, already known since *Cassie and Baxter 1944”, culminating in a reviewer´s comment *“against textbook knowledge, the so-called Lotus Effect exists only in the fantasy of the authors”*. When the manuscript was submitted to Planta in 1996, it was also rejected, but the Editor-in-Chief Andreas Sievers had followed our experiments, and after his intervention, the paper was published. The mechanism of self-cleaning was described *“…the interdependence between surface roughness, reduced particle adhesion and water repellency is the keystone in the self-cleaning mechanism of many biological surfaces…and may be of great technological importance*” (Barthlott and Neinhuis 1997). Particle removal with water was experimentally tested, even using the hydrophobic Sudan IV-powder (Fig. 2a, Barthlott 1992), forming structures later called liquid marbles (Aussillous and Quéré 2001). For SEM images, water was replaced in 1997 by small mercury droplets (Fig. 2b).

The publication in Planta in 1997 caused immediate wide attention, but also skepticism—mainly by industrial companies with competing technologies for antiadhesive flat surfaces, and physicists claimed the lack of theoretical modeling to explain the self-cleaning effect. Until today, it is not fully understood: *“Despite the enormous interest in superhydrophobicity for self-cleaning, a clear picture of contaminant removal is missing”* (Geyer et al. 2020). Even less understood are derivatives of the Lotus Effect, like the paradoxical Salvinia Effect (Barthlott et al. 2010; Gandyra et al. 2020). A few details concerning the Lotus discovery are mentioned in Forbes (2005, 2008), Barthlott (2014), Neinhuis (2017), and in Fujishima et al. (2023).

The 1997 article became one of the two most cited publications in the 100 years history of Planta, resulting in more than ten thousand follow-up publications (White 2018; Vonna 2023). It led to a “*paradigm shift in surface science”* and is sometimes considered as *“the most famous inspiration from nature … widely applied…in our daily life and industrial productions”* (Yu et al. 2020). It generated attention from the industries and in the scientific community, starting with Von Baeyer (2000), Li et al. (2000), Marmur (2004), Otten and Herminghaus (2004).

The seemingly old-fashioned taxonomic research of the author has been connected to the huge living plant collections at the Universities of Heidelberg, Berlin and Bonn. Our earliest SEM study (Barthlott and Ehler 1977) was already based on living material of about 2000 plant species; four decades later, a comprehensive survey and review (Barthlott et al. 2017) was based on more than 20.000 species examined by SEM, atomic force microscopy, and other techniques. Over 200.000 SEM micrographs of biological surfaces produced since 1971 were archived in Bonn in 2022. After half a century of research in biological surfaces, this personal summary presents the experience, publication history, patenting difficulties, and some still open questions. A historic perspective of superhydrophobicity will be provided in a subsequent publication.

## Physics, materials science, and plant surfaces

Wettability is measured by the contact angle of a water droplet on a surface (Law 2014). Extreme water repellency or superhydrophobicity is an effect of incomplete wetting by air trapped on the surfaces, caused by their nanoscopic to microscopic structures. It is usually defined by a static contact angle of water droplets of > 140°and reaches > 165° in plant surfaces like in *Nelumbo* (Ensikat et al. 2011).

The fundamentals of wetting of flat surfaces date back to Young (1805). Extreme water repellency had been noticed already by Aristotle in the fern *Adiantum* and gained interest from the eighteenth century on in *Lycopodium* powder. Trapped air as the physical background for superhydrophobicity was first described by Ziegenspeck (1934, 1942). Textile engineers noticed this effect of biological porous surfaces in industrial research: Wenzel (1936) based on “woven or knitted fabrics”, similarly Cassie and Baxter (1944) in their theoretical framework for heterogeneous surface wetting of textiles. The equations were later refined in non-biological materials in context with polytetrafluorethylene (PTFE) technologies (e.g., Johnson and Dettre 1964), but details are still disputed (Murakami et al. 2014; Shardt and Elliot 2018). Self-cleaning is bound to defined dimension of the hierarchical structure (Fig. 1d–f) and is absent, e.g., in too large hairy structures like the indumentum of plants or mammals: the textile Wenzel–Cassie–Baxter surfaces are superhydrophobic, but in contrast to Lotus leaves not self-cleaning.

Water on powders and dust forms particle-coated droplets or liquid marbles (Fig. 2a), e.g. on *Lycopodium* powder, and their high contact angles were first measured by Hélois Ollivier (“Recherches sur la capillarité”, thesis Sorbonne 1907). Hydrophobic powders were in focus since 1964: smoked silica (patent DE1964D0043745) generating liquid marbles (Fig. 2) and dry water (McHale and Newton 2011). In this paper, we use the term “conological” (from Greek *konis/konē*, meaning dust or fine powder) to describe powder- and dust-related effects, which are currently underrepresented in scientific research. Even hydrophilic powders can generate short-lived hydrophobic repellency like ephemerous water droplets on dry wheat flour.

**Fig.4:**
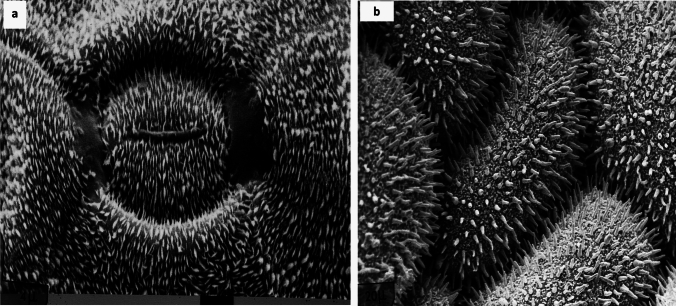
Two enigmatic superhydrophobic surfaces still pose open questions. **a** Wax platelets surrounding a stoma of the lily-of-the-valley (*Convallaria majalis*) are arranged in the pattern of electromagnetic field lines by an unknown electrophysiological process. Picture available at https://www.flickr.com/photos/lotus-salvinia/11081584045/in/album-72157638107887554. **b** The massive dimorphic rodlets on the seed coat surface of *Sceletium (Mesembryanthemum) tortuosum* are self-assembling and look like wax crystals, but they are resistant to a treatment with organic solvents or acids like sporopollenin. This is of interest to chemists and material science as an example of self-cleaning surfaces of high stability. Picture available at https://www.flickr.com/photos/lotus-salvinia/11081629295/in/album-72157638107329975

The chemistry of the surface plays a subordinate role for water repellency compared to the crucial spatial structures (Fig. 1d, e, Fig. 4), which range from nanometer to millimeter scale. The epicuticular morphologically complex wax covers of plants (Barthlott et al. 1998) are chemically highly diverse (Zeisler-Diehl et al. 2020)

*Nelumbo* self-assembling tubular crystals (Fig. 1e, f) of the secondary alcohol nonacosan-10-ol with a diameter of 110 nm and a length of 1–2 µm (Koch et al. 2004, 2009c; Dora et al. 2018) are responsible for the wax structures. They are superimposed onto cellular structures from micrometer (e.g. papillate cells) to millimeter (e.g., trichomes) range (Fig. 1d, Fig. 4) (Barthlott et al. 2017).

Nano- and microstructures erode under the influence of water and other atmospheric factors. Superhydrophobicity is a metastable state (Marmur 2004), a highly dissipative system far from a thermodynamic equilibrium, and its maintenance requires a constant decrease of entropy. It is usually bound to living organisms and more or less absent in abiotic natural materials, where only contact angles occur up to about 100°, e.g., in flat carbon (graphite, Yasuda 1994). However, very small instable particles like soot or carbon nanotubes are superhydrophobic (Babu et al. 2017). In artificial fluoropolymer flat surfaces, contact angles reach up to 120°, in structured PTFE about 165° (Burkarter et al. 2007).

Terminological confusion exists. *Hydrophobia* is a late medieval term for rabies (water shyness), *hydrophobicity* for water repellency arose only very late in the second decade of the twentieth century. The term *superhydrophobicity* seems to be first used in 1974 in a patent application (US Patent # 3,931,428), the superfluous term *ultra-hydrophobicity* appears in publications since 1999. For *superhydrophobic and self-cleaning* surfaces, the term *Lotus Effect* was introduced in 1992. Self-cleaning, but hydrophilic surfaces, became important in the different photocatalysis technology since the 1990 s (Fujishima et al. 2023).

**Fig. 5:**
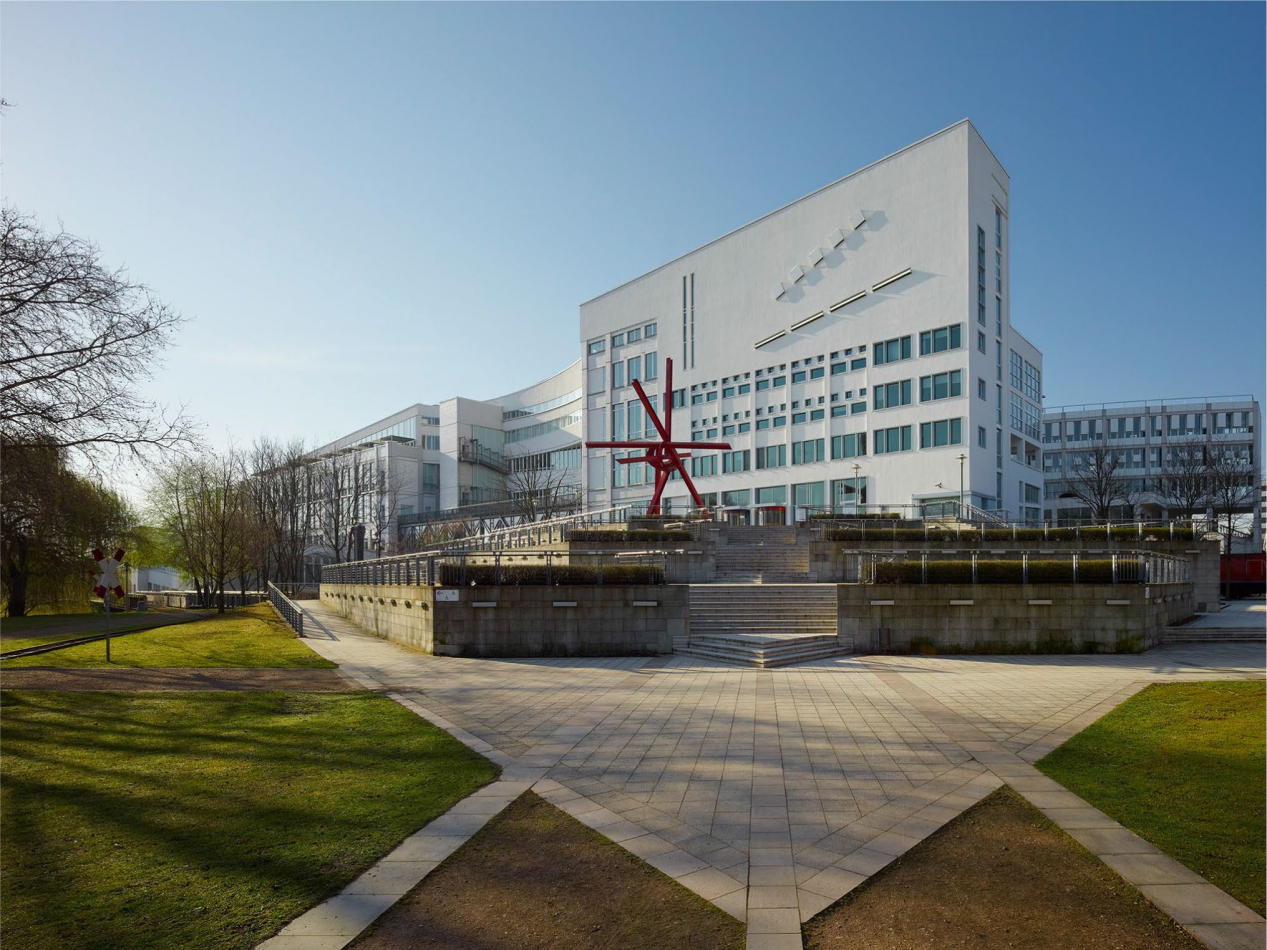
First technical Lotus Effect products came on the market in 2001. One of the most successful applications are façade paints: above the Technoseum in Mannheim, a major German technical museum, coated in 2010 with a self-cleaning Lotus Effect paint (*Sto Lotusa*n). Foto Technoseum, Fig. 3.12 from Barthlott et al. (2016b)

Surface interactions are highly complex. The physicist Hans Christian von Baeyer entitled enthusiastically a paper: “*The Lotus Effect: The secret of the self-cleaning leaves of the lotus plant, like the subtlest applications of high technology, is simplicity itself”* (Von Baeyer 2000)—but superhydrophobicity and self-cleaning are still not fully understood (e.g., Drelich et al. 2019; Geyer et al. 2020). Biological surfaces played no role in the 1960 rediscovery of nature inspired technology under the new name *Bionics* in the Dayton-report (Barthlott et al. 2016b). Half a century later, surface science and nanotechnology became emerging fields (Bhushan 2017; Guo and Yang 2017; Yu et al. 2020; Sotoudeh et al. 2023) and biomimetics is an important subject today (Bhushan 2018). Superhydrophobic surfaces received much attention for application across industries (Fig. 3b, Fig. 5) (Barthwal et al. 2024).

### Superhydrophobic abiotic and biological surfaces: occurrence and evolution

Superhydrophobicity seems to be absent in abiotic natural surfaces apart from the usually short-lived unstable water repellency caused by “conological” effects of dust and powders, independent from their chemical property. This effect is caused by particle-coated droplets (Fig. 2a) and instable, but important in industrial processes associated with dust, powders, or in mining, and a globally increasing problem of temporarily non-wettable soils (Majid et al. 2025). Small structured soil particles are often caused by the influence of mechanical micro-erosive structuring under insolation or by fire (DeBano 2000; Then et al. 2025) generating fumed silica particles similar to industrial powders (Forny et al. 2009). Soil unwettability is not necessarily caused by organic compounds or microorganisms. A reversible transition of states is seen in peat (e.g., of *Sphagnum*): superhydrophobic when dry, water splashes off when watering a dry peat bale or organic soil, but eventually it turns superhydrophilic absorbing water (Perdana et al. 2018).

Biotic superhydrophobic surfaces in animals are very common in many terrestrial taxa**,** e.g., in arthropods (Wagner et al. 1996) or bird feathers. It allows the ability to fly in rain or to dive under water with reduced friction (passive air lubrication). Self-cleaning plays a minor role in animals apart from certain insects, because they are usually able to clean their surfaces actively. Insect cuticles are particularly well researched (Gorb 2001). Superhydrophobicity enables persistent air layers (“plastron”) under water, which were studied in plants (e.g., Barthlott et al. 2010; Gandyra et al. 2020) and in insects (e.g., Wagner et al. 1996; Mail et al. 2018).

### Green plants

Dust effects play a role in mobile spores, pollen, and dust seeds of the sessile angiosperms. These small dispersal units characteristically show multifunctional surface sculpturing (Rauh et al. 1975), e.g., to increase the Reynold numbers for floating in the air, or for attachment and detachment. Upon landing on a water surface, they may float several weeks—but in some cases, they become wettable within minutes—even the whole surface morphology changes as in certain dust seeds like the orchid *Chiloschista* (Barthlott et al. 2014). Plants evolved conological effects mainly as a dispersal mechanism in spores or wind dispersed pollen.

Sessile plants interact in contrast to mobile animals with their environment by a chemical arsenal of secondary metabolites over their surfaces. Their hydrophobicity is caused by chemically and morphologically most diverse epicuticular crystals (Fig. 1d–f, Fig. 4) (Barthlott et al. 1998; Zeisler-Diehl et al. 2020), which are self-assembling (Koch et. al. 2004; Ensikat et al. 2006). Wax platelets can show parallel orientations for anisotropic wetting effects in monocotyledons (e.g., in rice), even oriented in the pattern of magnetic field lines (Fig. 4a). Wax crystals are hierarchically superimposed on the differing epidermal cell shapes (Fig. 1d–f, Fig. 4b) from flat, papillate to trichomes. Under mechanical or atmospheric influence, waxes erode and old leaves become less hydrophobic or even hydrophilic. Rather stable, but less efficient, are the self-assembling polymerous folding pattern of the cuticle itself (Barthlott 1980). They are characteristic for surfaces of flowers, because wax crystals reduce the attachment of legs of insect pollinators like in traps of insectivorous plants (*Nepenthes, Sarracenia*): only flowers pollinated by birds are often heavily covered by wax (e.g., Chilean bellflower *Lapageria*). Simultaneous occurrence of wax crystals and cuticular folds is not functional and was not observed.

Damaged epicuticular wax regenerates within very different times (from minutes to days, Koch et al. 2004), but some species are not able to regenerate (e.g., the succulent *Dudleya farinosa*, Crassulaceae). Wettable cuticular surfaces occur frequently for water uptake in epiphytes (e.g., Bromeliaceae, Orchidaceae) or in plants of fog-deserts like in the Chilean cactus *Copiapoa cinerea* with thick water absorbing wax crusts. Submersed water plants are hydrophilic, but can exhibit enigmatic effects, like on the leaf surfaces of the pondweed *Potamogeton*, which is hydrophilic submerged in water and water droplets roll off when floating on the water surface.

### Evolution of superhydrophobicity

In non-phototrophic organisms, superhydrophobicity occurs in the capillitium and spores of slime molds and reproductive structures of fungi and lichens, in Collembola, and virtually in all insect groups and many terrestrial vertebrates (Barthlott et al. 2022). Data from fossils are not available; however, there are other indicators: the giant dragonfly *Meganeura* in the late carboniferous with thin wings (Barthlott et al. 2016b) was hardly functional without a superhydrophobic surface similar to all modern Odonata (Wagner et al. 1996). Land plants evolved since the Middle Cambrian approximately 400 Mya. In non-aquatic green plants, superhydrophobicity is known from all groups from bryophytes to angiosperms (Barthlott et al. 2020). Most common are the tubular crystals of nonacosan-10-ol, but certain complex crystal shapes circumscribe whole orders of monocotyledons and of some woody ancestral families (Barthlott 1990): The trait, thus, appears to be conserved, indicating a stable and ancient genetic anchoring. Reversible superhydrophobicity occurs in terrestrial biofilms of some cyanobacteria (*Hassallia*) and green algae indicating a much earlier origin. Possibly superhydrophobicity was a key evolutionary step over a billion years ago in the land-transition of Precambrian autotrophic organisms (Barthlott et al. 2022).

### Global extension of superhydrophobicity

We estimated that about 90% of all green plants are at least partially superhydrophobic, like grasses (Poaceae). Grasslands are among the largest ecosystems in the world covering about 50 million km^2^ (remote sensing data). If we assume that one m^2^ grassland has a cuticular surface of at least ten m^2^, the global area of superhydrophobic cuticular grass surfaces comprises at least 500 million km^2^. Cuticular surfaces are possibly the largest homogenous interfaces between solids and the atmosphere on our planet.

## Conclusion and perspectives

The publication of plant’s self-cleaning superhydrophobic surfaces in Planta (1997) led to a paradigm shift in surface sciences. Until 1998, only flat surfaces were industrially applied for dirt-repellent materials, e.g., PTFE. In the first visits, in industrial laboratories from 1992 on, we learned that exclusively “*flat"* means clean, and *“rough*” was out of imagination and that the biological effect of Lotus was not transferable to materials. Only after having generated the first biomimetic prototype in 1994 (Fig. 3b), industrial cooperation began (Forbes 2005, 2008) and the research gained worldwide attention. The broad range of applications was early evident (Yan et al. 2011), today focusing on paints, coatings, certain textiles, and sprays. A well-known example is the sustainable self-cleaning façade paint “Lotusan” (Fig. 5).

The discovery had led to a “creative destruction” (J. Schumpeter) of certain established technologies and was bound to meet rejection (Barthlott 2014). The University of Bonn had encouraged us already in 1994 to file a patent application: “*Self-cleaning surfaces of objects and processes for producing the same*”. In 1997, the trademark *‘Lotus-Effekt*^*®*^*’* and later *‘Lotus-Effect*^*®*^*’* were registered. The application caused immediate attention of the industries and first successful co-operations, but also led instantly to conflicts. The wrong equation superhydrophobicity = self-cleaning played an important role, claiming the Lotus Effect was described already by Cassie and Baxter in 1944, and e.g., patented in the US by DuPont in 1952. It is stimulating to read Martin (2007) concerning scientists, patents, and industries. The European Patent was finally revoked in 2010—after 12 years of fighting oppositions from the industry, but the trademarks are still in force and now owned and used by STO SE & Co, KgaA (Stühlingen, Germany).

The Lotus Effect led to the discovery of several related phenomena like the Petal Effect, the superhydrophilic and oleophilic surfaces of *Ruellia* (Koch et al. 2009a, b, c) to the discovery of the Salvinia Effect (Barthlott et al. 2010; Gandyra et al. 2020), e.g., for underwater drag reduction by passive air lubrication (Busch et al. 2019). Air-retaining surfaces provide the potential for different applications, e.g., the removal of oil films from water surfaces (Barthlott et al. 2020) or underwater pressure sensing (Mail et al. 2018). The Lotus Effect and its Salvinia Effect derivative have changed different aspects of surface technologies up today.

In the new millennium, bionic and biomimetic technologies have become increasingly important (Barthlott et al. 2016b) and relevant to everyday applications. Networks were established like BIOKON in Germany in 2001, the International Society of Bionic Engineering ISBE in 2010, and in 2025 the International Academy of Bionic Science (ABS). Important work is currently being carried out in China. Surface science is still a young field and in plant science probably underestimated—and still many open questions remain. Cuticles provide probably the largest rather homogenous surface area on our planet—possibly exceeding that of the oceans—and play a crucial role, even in understanding climate change. Apart from our assessments of the global area of cuticular surfaces (e.g., Barthlott et al. 2017), no thorough assessment of its dimension seems to be available. These surfaces influence the radiative cooling performance of the vegetation cover, but the leaf area index is not sufficient to understand the interface effects. We drew repeated attention to a possible cooling effect of surfaces under insolation as a result of hierarchical structuring (Barthlott 1981, 1990; Porembski et al.^1992^; Barthlott et al. 2017), but we could not provide experimental data. Only recently the radiative cooling performance in the context of insolation and insulation was proven in self-cleaning structured technical surfaces (Li et al. 2025; Long et al. 2025), which should stimulate further botanical research.

To conclude and to encourage further research, a few examples. Not understood are the wax platelets strictly oriented in an electromagnetic field pattern crossing many single cells surrounding the stomata in *Convallaria majalis* (Fig. 4a) and related monocotyledons (Barthlott and Frölich 1983; Barthlott et al.^1998^). The chemistry of the massive rodlets on the seed coats of certain Aizoaceae like in *Sceletium tortuosum* is still unknown: self-assembling crystals (Fig. 4b), but resistant to a treatment with organic solvents or acids like sporopollenin (Ehler and Barthlott 1978); thus being an interesting structure for chemists and material scientists to fabricate self-cleaning surfaces of high stability.

Finally, the Lotus Effect in plants is the first defense barrier against pathogen attachment, but still unsolved is its fate after application of surfactants, which are among the most widespread chemicals added in agriculture to allow wetting and penetration of pesticides. *“There is strong evidence that the commercial application of surfactants increases the susceptibility of plants to pathogenic micro-organisms*” (Barthlott 1990). We could show that the application of surfactants, which is essential in agriculture, enhances the adhesion, e.g., of fungal spores, and thus the defense mechanism is reduced (e.g., Noga et al. 1987; Wolter et al. 1988; Neinhuis et al. 1992; Schwab et al. 1995). The application, formulation, and role of surfactants in crop protection need to be reconsidered. A final recommendation for further critical and courageous research for surface science application: organismic biologists should continue their taxonomic research to explore and conserve the still very insufficiently known, but disappearing knowledge on biological diversity with many potential bionic “role models” for future applications.

## Data Availability

Not applicable.
